# Deleterious oral habits related to vertical, transverse and sagittal dental malocclusion in pediatric patients

**DOI:** 10.1186/s12903-022-02122-4

**Published:** 2022-03-23

**Authors:** Lourdes Hilda Gabriela Rodríguez-Olivos, Pamela Roxana Chacón-Uscamaita, Antony Germán Quinto-Argote, Graciela Pumahualcca, Luis Fernando Pérez-Vargas

**Affiliations:** 1 School of Dentistry, Universidad Nacional Mayor de San Marcos, Lima, Peru; 2 Emerge, Emerging Diseases and Climate Change Research Unit, School of Public Health and Administration, Universidad Peruana Cayetano Heredia, Lima, Peru

**Keywords:** Habits, Malocclusion, Children

## Abstract

**Background:**

Malocclusion is highly reported among mixed dentition cases. Therefore, we aimed to determine the relationship of dental malocclusions in the vertical, transverse, sagittal planes with deleterious habits in pediatric patients.

**Methods:**

A cross-sectional analytical study was carried out on 155 children aged 6–12 years attended at the clinic of the School of Dentistry of Universidad Nacional Mayor de San Marcos in 2017.

**Results:**

Among 155 evaluated patients, 45.3% had vertical malocclusion, 52.0% had sagittal malocclusion and 13.6% had transverse malocclusion. The most frequent type of malocclusion in the vertical plane was anterior deep bite (22.2%), in the transverse plane, the edge-to-edge bite (7.1%) and the anterior crossbite (6.5%) were less frequent. Finally, in the sagittal plane, Class II Div 1 (20%) and Class III (20.7%) were the most frequent. Among the most common deleterious habits, anteroposition (58.7%) and mixed breathing (51.0%) were observed in contrast to the habit of retroposition, lip sucking and mouth breathing, which were the least frequent. Considering age and sex, children who have an atypical swallowing habit are more likely to have malocclusion in all three planes of space.

**Conclusions:**

It is concluded that there is an association between the deleterious habits with the different types of malocclusions in the different planes of the space, being the atypical swallowing a habit that should be early diagnosed and treated interdisciplinary.

## Background

Malocclusion is a misalignment of the upper and lower teeth, where an adequate gear of bone structures between the maxilla and jaw is not observed, resulting on the lack of an ideal function of the masticatory apparatus [1]. This condition, which has a prevalence of 79.4% in children with mixed dentition [2, 3], is considered a public health problem because it causes psychological problems and affects quality of life [4, 5]. Malocclusions can be classified according to the three planes of space (vertical, transverse and sagittal), where vertical malocclusions are classified as open bite and deep bite [6]. The open bite is a condition which one or more teeth cannot contact their antagonists and is related to the mouth breathing habit when the airway obstruction is not resolved [7, 8]. In contrast, the deep bite has an increased vertical overbite [9] and boys have more overbite than girls [4].

Transverse malocclusions are disorders where the upper and lower posterior teeth do not occlude properly and are associated with prolonged sucking habits [10]. They are classified as unilateral posterior, bilateral posterior crossbite and scissor bite (Brodie bite) [11]. Sagittal malocclusion is classified according to Angle's classification into Class I, Class II and Class III malocclusion. Class I is where the mesial cusp of the upper first molar occludes between the mesial and median cusps of the first lower molar, Class II is when the permanent lower molar occludes distally from its normal position. Finally, Class III manifests the inversion of dental relationships, by the first permanent lower molar that is mesially occluded with respect to its normal position [12, 13].

Children experience a phase of growth and development in which their bone structures are moldable and physiological habits serve as stimuli for normal jaw growth (e.g., speech, normal swallowing, chewing). Deleterious habits (e.g. thumb sucking, lip sucking and biting, nail-biting, bruxism, mouth breathing and tongue thrusting) can interfere with the dental structure and may be part of the etiology of malocclusions that can cause an imbalance in muscle strength and changes in the normal functional aesthetics of the entire stomatognathic apparatus [14, 15]. Therefore, the failure to do so cannot be corrected without first addressing these reflex activities. There are few studies on the association of deleterious habits with sagittal, vertical and transverse dental malocclusion [16–20]; and it is still under discussion whether oral habits and mouth breathing are part of the etiology of malocclusions [21], however, the recent results of this research mention that bad habits and mouth breathing are risk factors for malocclusion [22], in addition, studying the cause-effect relationship between malocclusions and deleterious habits is relevant for an adequate intervention in the treatment and to obtain better results.

The aim of this study was to determine the relationship of dental malocclusions in the vertical, transverse, sagittal planes with deleterious habits present in patients aged 6 to 12 years attended at the clinic of the School of Dentistry of Universidad Nacional Mayor de San Marcos in 2017.

## Methods

### Study design and sample selection

A cross-sectional study was conducted, among a population of 260 children attending the Undergraduate Clinic of the School of Dentistry of Universidad Nacional Mayor de San Marcos (FO-UNMSM) in 2017. A probabilistic sampling was performed considering a confidence level of 95%, estimating a prevalence of malocclusion of 50% and accuracy of 5% obtaining 155 patients; a random selection was performed using the Epidat program, we worked with the list of numbers of medical records of patients.

### Eligibility criteria

Eligible patients aged from 6 to 12 years were excluded if received previous orthodontic treatment, had a systemic disease or not willing to agree to participate in the study were excluded.

### Examiner training and calibration

The clinical examination was performed by one examiner who was trained by a specialist in orthodontics and scored a concordance of Kappa Index > 0.8.

### Clinical data collection

To recognize the atypical swallowing habit, the patient was given a plastic cup half full of water and was asked to drink a sip of water, lower the cup and slowly pass the content in the mouth. In this process, it was observed the swallowing habit, making a grimace, the contraction of the orbicularis muscles of the lips, masseter or jaw. It was observed if there is water leakage or if there is the interposition of the tongue between the incisors when swallowing. Cheek retractors were also used to better observe the tongue thrusting, and a little water was introduced with an injector, the patient swallowed when indicated so it was clearly observed if there was tongue thrusting or not.

To evaluate the thumb sucking habit, all the fingers of the hands were observed to recognize if they were wet and/or wrinkled, callosities, irritative eczema, paronychia in the thumb or index finger. For lip sucking, the patient was observed for one minute to check if the patient sucks the lower lip in a resting state. To recognize the type of nasal and/or mouth breathing presented by each patient, a tape with some cotton and a mask cut in half with a napkin was used. For one minute, the patient's nasal breathing was evaluated by placing a tape on the septum of the nose and in each nostril some cotton threads, this allowed observing if there was a movement of the cotton in each nostril, if the cotton moved it indicated air circulation through the airways, if the cotton did not move it indicated obstruction of the airway. The mouth breathing was analyzed by placing the mask with the napkin at the level of the patient's mouth for one minute, the movement of the napkin was observed to see if there was air flow through the mouth. The habit of onychophagia was evaluated by careful observation of the fingernails recognizing the shape of the nail saw, in addition to the lesions on the free edge of the nail and whether or not it disappears, also with the presence of microtrauma in the nail bed. Patient posture was evaluated with the sternal malar relation, in which, by means of a large square and a 30 cm ruler, the patient was asked to stand in profile with a Frankfurt plane parallel to the floor to measure a line from the midpoint of the cheekbone of the face towards the midpoint of the jugular notch. If both coincide in a patient with an ortho position, the malar point can be up to 2 cm in front of the midpoint of the jugular notch, if it exceeds this measure the head is in anteroposition. If the malar point is behind the midpoint of the jugular notch, the head is in retroposition. An analysis of the dentoalveolar malocclusion presented by the patient was performed: Sagittal dental malocclusion with Angle's classification (Class I, Class II Div 1, Class II Div 2 and Class III). Vertical dental malocclusion was evaluated according to the overbite. The deep bite was evaluated if there was an excessive overbite (> 20%). In an anterior open bite one or more teeth do not reach the occlusion line and do not make contact with antagonists (null overbite or negative). A transverse dental malocclusion evaluated the unilateral or bilateral posterior crossbite according to the vestibular cusps of the lower premolars and molars that laterally overbite the upper ones in one or both hemiarches; and scissor or Brodie bite according to the palatal faces of the upper molars and premolars contacting the vestibular faces of the lower teeth. In addition, the chronological age and gender of the child were recorded.

### Data analysis

For the statistical analysis a significance level of *p* < 0.05 was considered. Descriptive measures of absolute and relative frequencies are presented for categorical variables. A bivariate analysis was performed to evaluate malocclusions and deleterious habits according to sex, in addition to the association of deleterious habits with the different types of malocclusions in the three planes using the Chi-square test and Fisher's Exact Test. Generalized linear models with Poisson distribution and robust variance adjustments were used to estimate the prevalence ratios of having transverse, sagittal and vertical malocclusion according to the presence of deleterious habits, adjusting for age and sex. All analyses were performed with the Stata v.16 statistical package.

### Ethical statement

Approval was obtained from the FO-UNMSM Research Committee (N°110/FO-VDAC-AYOE). Patients signed consent and their parents informed consent. The research presented complies with all ethical statements in humans according to the Declaration of Helsinki.

## Results

From 155 evaluated patients, 71% had some type of malocclusion, 45.3% (n = 57) had vertical malocclusion, 52.0% (n = 75) had sagittal malocclusion and 13.6% (n = 21) had transverse malocclusion. According to Table 1, the most frequent type of malocclusion in the vertical plane was anterior deep bite (22.2%), in the transverse plane, edge-to-edge bite (7.1%) and anterior crossbite (6.5%) were less frequent. Finally, in the sagittal plane, Class II Div 1 (20%) and Class III (20.7%) are frequent. Among the most common deleterious habits, anteroposition (58.7%) and mixed breathing (51.0%) were observed in contrast to retroposition habit, lip sucking and mouth breathing which were the least frequent. In both sexes, the most prevalent deleterious habits were anteroposition and mixed breathing, and as for malocclusion according to the plane, they were anterior deep bite and Class III malocclusion for men and Class II Div 1 for women.

**Table 1:** Characteristics of patients aged 6 to 12 years attended at FO-UNMSM

	Total (N = 155)	Men (N = 75)	Women (N = 80)	*p*
Deleterious habits				
Atypical swallowing	51 (32.9%)	23 (30.7%)	28 (35.0%)	0.566*
Onychophagia	48 (31.0%)	23 (30.7%)	25 (31.3%)	0.937*
Thumb sucking	18 (11.6%)	8 (10.7%)	10 (12.5%)	0.722*
Lip sucking	8 (5.2%)	3 (4.0%)	5(6.3%)	0.720^†^
Mouth breathing	10 (6.5%)	3 (4.0%)	7 (8.8%)	0.330^†^
Mixed breathing	79 (51.0%)	42 (56.0%)	37 (46.3%)	0.225*
Anteroposition	91 (58.7%)	43 (57.3%)	48 (60.0%)	0.736*
Retroposition	7 (4.5%)	3 (4.0%)	4 (5.0%)	1.000^†^
Any Malocclusion	110 (71.0%)	48 (64.0%)	62(77.5%)	0.064*
- Vertical**				
Normal	69 (54.8%)	35 (56.5%)	34 (53.1%)	
Anterior open bite	12 (9.5%)	4 (6.5%)	8 (12.5%)	0.711*
Edge-to-edge bite	17 (13.5%)	9 (14.5%)	8 (12.5%)	
Anterior deep bite	28 (22.2%)	14(22.6%)	14 (21.9%)	
- Transverse				
Normal	133 (86.4%)	64 (85.3%)	69 (87.3%)	
Edge-to-edge bite	11 (7.1%)	5 (6.7%)	6 (7.6%)	0.750*
Posterior crossbite	10 (6.5%)	6 (8.0%)	4 (5.1%)	
- Sagittal				
Class I	75 (50.0%)	38 (52.1%)	37 (48.1%)	
Class II Div 1	30 (20.0%)	11 (15.1%)	19 (24.7%)	0.359*
Class II Div 2	14 (9.3%)	9 (12.3%)	5 (6.5%)	
Class III	31(20.7%)	15 (20.6%)	16 (20.8%)	

*Chi-Square Test. ^†^Fisher's Exact Test

**May add up to less than 155 for unrecorded values

Deleterious habit of atypical swallowing is statistically associated with different types of vertical dental malocclusion (*p* < 0.001), 91.7% of children with atypical swallowing presented edge-to-edge bite (11 of 12) (Table 2). Also, different types of transverse dental malocclusion are associated with mouth breathing (*p* = 0.001) and mixed breathing (*p* = 0.043), as can be seen in 7 of the 10 patients with mixed breathing presented posterior crossbite (Table 3). There is an association with the types of sagittal malocclusion with the habits of atypical swallowing (*p* = 0.002), lip sucking (*p* = 0.039), anteroposition (*p* = 0.006) and retroposition (*p* = 0.010) (Table 4).

**Table 2:** Deleterious habits by type of vertical dental malocclusion in patients aged 6 to 12 years

Present	Normal (n = 69)	Edge-to-edge Bite (n = 12)	Anterior open bite (n = 17)	Anterior deep bite (n = 28)	*p*
Atypical swallowing	14	11	7	10	< 0.001*
Onychophagia	23	1	6	7	0.305*
Thumb sucking	5	2	3	5	0.366*
Labial sucking	3	1	0	3	0.389^†^
Mouth breathing	3	2	0	2	0.214^†^
Mixed breathing	36	9	9	9	0.080*
Anteroposition	41	9	7	15	0.305*
Retroposition	3	1	2	1	0.444^†^

*Chi-Square Test. † Fisher's Exact Test

**Table 3:** Deleterious habits by type of transverse dental malocclusion in patients aged 6–12 years

	Normal (n = 133)	Edge-to-edge bite (n = 11)	Posterior crossbite (n = 10)	*p*^†^
Atypical swallowing	40	7	4	0.070
Onychophagia	39	5	4	0. 411
Thumb sucking	15	2	1	0.748
Labial sucking	6	2	0	0.183
Mouth breathing	4	3	3	0.001
Mixed breathing	69	2	7	0.043
Anteroposition	79	7	5	0.877
Retroposition	4	1	1	0.189

^†^Fisher's Exact Test

**Table 4:** Deleterious habits by type of sagittal dental malocclusion in patients aged 6–12 years

	Class I (n = 75)	Class II Div 1 (n = 30)	Class II Div 2 (n = 14)	Class III (n = 31)	*p*
Atypical swallowing	15	17	4	13	0.002*
Onychophagia	24	8	4	11	0.893*
Thumb sucking	9	1	4	3	0.118^†^
Labial sucking	2	5	0	1	0.039^**†**^
Mouth breathing	3	4	0	0	0.088^†^
Mixed breathing	38	18	4	17	0.265*
Anteroposition	48	22	8	10	0.006*
Retroposition	1	0	0	5	0.010^**†**^

*Chi-Square Test. ^†^Fisher's Exact Test

Children with atypical swallowing habit are more likely to have malocclusion in all three planes of space (vertical (PR = 1.90, CI 95%: 1.31–2.74), sagittal (PR = 1.68, CI 95%: 1.26–2.25) and transverse (PR = 2.28, CI 95%: 1.04–5.01). In addition, children with thumb sucking are more likely to have vertical malocclusion (PR = 1.54, CI 05%: 1.00–2.38), children with mouth breathing are more likely to have transverse malocclusion (PR = 6.15, CI 95%: 2.96–12.8) and children with retroposition are more likely to have sagittal malocclusion (PR = 1.65, CI 95%: 1.13–2.42) (Table 5).

**Table 5:** Deleterious habits associated with the presence of malocclusion in patients aged 6 to 12 years

	Malocclusion vertical (n = 126) PRa (CI95%)	Malocclusion sagittal (n = 150) PRa (CI95%)	Malocclusion transverse (n = 154) PRa (CI95%)
Atypical swallowing	1.90 (1.31–2.74) *p* = 0.00﻿1	1.68 (1.26–2.25) *p* < 0.001	2.28 (1.04–5.01) *p* = 0.040
Onychophagia	0.79 (0.50–1.26) *p* = 0.325	0.97 (0.69–1.36) *p* = 0.870	1.66 (0.75–3.67) *p* = 0.214
Thumb sucking	1.54 (1.00–2.38) *p* = 0.049	1.15 (0.75–1.76) *p* = 0.517	1.29 (0.42–3.94) *p* = 0.660
Labial sucking	1.26 (0.63–2.54) *p* = 0.512	1.47 (0.96–2.26) *p* = 0.079	1.94 (0.54–6.87) *p* = 0.307
Mouth breathing	1.22 (0.60–2.47) *p* = 0.579	1.09 (0.56–2.10) *p* = 0.809	6.15 (2.96–12.8) *p* < 0.001
Mixed breathing	0.93 (0.63–1.38) *p* = 0.729	1.11 (0.82–1.52) *p* = 0.504	0.71 (0.31–1.59) *p* = 0.399
Anteroposition	0.89 (0.61–1.30) *p* = 0.542	0.82 (0.60–1.11) *p* = 0.200	0.93 (0.41–2.08) *p* = 0.854
Retroposition	1.26 (0.63–2.55) *p* = 0.508	1.65 (1.13–2.42) *p* = 0.010	2.67 (0.87–8.24) *p* = 0.087

PRa: Prevalence ratios (CI 95% Confidence Intervals) adjusted with the sex and age variable

## Discussion

Thumb and lip sucking habits are complex neuromuscular patterns considered normal in childhood and abnormal from 3 years of age [15]. The persistence of these habits can affect dentofacial growth [1], therefore prevalence research in transversal and longitudinal evaluation allows us to understand their impact on child growth and development.

Research in Nigeria [16], Cuba [17], Peru [18] and Italy [19] found that there was a direct relationship between deleterious habits and malocclusion, as in our study which found a relationship between the habit of atypical swallowing, thumb sucking, mouth breathing and retroposition with the presence of malocclusion. However, contrary to the Italian study, our study did find a relationship between deleterious habits and malocclusion types. Atypical swallowing and mouth breathing were related to vertical malocclusion, mixed breathing related to transverse malocclusion, and atypical swallowing, labial sucking, anteroposition related to sagittal malocclusion. In Spain, another author shows that deleterious habits can initiate, predispose and aggravate dental malocclusions, although they are not the main etiological factor governing their appearance [20].

Furthermore, according to Fialho and Col. [23] there is a relationship between non-nutritive sucking habits and anterior open bite, and concluded that the presence of deleterious habits was not determinant for facial morphology. That is why we recommend analyzing facial morphology and looking for an association with deleterious habits. In this study we understand that there are intrinsic factors that can be recognized by the dentist and there are also extrinsic factors (genetics) that can act in isolation or in combination leading to malocclusions.

This study shows the existence of a statistically significant relationship between anterior deep bite with the mixed breathing habit, anterior open bite with the atypical swallowing habit. Jamilian showed that children with the habit of thumb sucking have a greater risk of suffering anterior open bite and posterior crossbite [5], coinciding with the research carried out in Spain [24] and India [25].

It is important to mention that bottle-feeding during weaning is a risk factor, not evaluated in this study, which was strongly associated with anterior open bite, according to the longitudinal study conducted by Moimaz et al. [26], who monitored sucking habits and nocturnal oral breathing from pregnancy to 30 months of birth.

Our results corroborated the findings of Condori et al. [27], showing that there is a statistically significant association of transverse malocclusion, such as posterior crossbite, with mouth breathing habit; edge-to-edge bite (transverse) with atypical swallowing habits; mouth breathing and mixed and scissors bite with retroposition posture habit.

In addition, a study in adolescents in Northeastern Brazil showed that deleterious habits cause premaxilla conditions, protrusion of the upper incisors causing anterior open bite and posterior crossbite [28]. The prevalence of posterior crossbite is caused by poor oral habit leading to low tongue positioning during sucking, lack of tongue thrust to the palate mainly causes increased activity of the cheek muscles. This, in turn, leads to altered muscle pressure in the upper arch, resulting in malocclusion. [29].

It should be noted that Condori et al. [27] added an anatomical factor to the research and found a direct relationship with maxillofacial alterations, according to the degree of adenoid obstruction caused by adenoid hypertrophy. Likewise, Rossi et al. [30] defined a direct relationship between the degree of nasal obstruction and its repercussion on the facial, skeletal and dental pattern. However, a strong evidence-based association has not yet been established [31].

The Class I malocclusion without anterior crossbite was associated with atypical swallowing, however atypical swallowing does not generate a Class I malocclusion but rather, this habit is present in open bites and in Class II malocclusions according to Jimenez [18], this association may be due to the fact that our sample has a Class I skeletal pattern and there has not been a transition of the swallowing pattern, they remain with childlike swallowing. The Class I malocclusion with anterior crossbite was related to thumb sucking habit, coinciding with a research paper carried out in Brazil [32]. This may be due to a thumb sucking in a horizontal position of the fingers that stimulates a forward sliding of the jaw, just as Jimenez [18] we found that Class II Div 1 malocclusion was related to atypical swallowing and lip sucking habits (*p* < 0.05). It was found in the research in agreement with the aforementioned authors that there is no statistically significant association of Class II Div 2 malocclusion with deleterious habits, one possibility is that this malocclusion is influenced by genetics and not by habits.

Class III malocclusion without anterior crossbite was associated with anteroposition posture, the individuals in the sample presented a Class III dental malocclusion that could be due to loss of lower teeth that caused mesialisation of the lower first molars, however, they have a Class I or II skeletal pattern. Class III malocclusion with anterior crossbite had a statistically significant association with retroposition posture. This is explained because in children with Class III malocclusion the angle of cervical lordosis is lower than in children with Class I and Class II malocclusion [33].

Among the limitations that arose were not registering Graber’s trident with which the intensity, duration and frequency of each deleterious habit can be evaluated and according to that relate it to skeletal changes. In addition, it is recommended to use an index of need for orthodontic treatment for the diagnosis of malocclusions; and since it is a cross-sectional study, it is not possible to know if the habits appeared after having malocclusion. It is not possible to extrapolate the data to all pediatric patient, because the population was children who come to a School of Dentistry in need of care. It is recommended to evaluate vertical, transverse and sagittal malocclusions with vertebral defects and find their correlation. It is also recommended to evaluate age ranges to investigate in which the deleterious habit generates greater malocclusion and in which type of dentition.

## Conclusions

It is concluded that there is an association between deleterious habits and the different types of malocclusions in the different planes of space, being atypical swallowing a habit that should be treated interdisciplinary and referenced from its diagnosis to reduce the probability of the presence of malocclusions. The most prevalent deleterious habits were anteroposition and mixed breathing, and as for malocclusion according to the plane, they were anterior deep bite for both sexes and Class III malocclusion for men and Class II Div 1 for women.

## Data Availability

The datasets used and/or analyzed during the current study are available from the corresponding author on reasonable request.

## Abbreviation

FO-UNMSM: Undergraduate Clinic of the School of Dentistry of Universidad Nacional Mayor de San Marcos

## References

[1] Vellini F, Hecht M (2002). Oral habits in orthodontics. Orthodontics, diagnosis and clinical planning.

[2] Yu X, Zhang H, Sun L, Pan J, Liu Y, Chen L (2019). Prevalence of malocclusion and occlusal traits in the early mixed dentition in Shanghai, China. PeerJ..

[3] Barbosa TS, Gavião MBD (2008). Oral health-related quality of life in children: part II. Effects of clinical oral health status. A systematic review. Int J Dent Hyg..

[4] Vieira-Andrade RG, Marques SM de P, Marques LS. Impact of malocclusions on quality of life from childhood to adulthood. In: Bourzgui F, editor. Issues in contemporary orthodontics. Croatia: IntechOpen; 2015. p 39–55.

[5] Jamilian A, Kiaee B, Sanayei S, Khosravi S, Perillo L (2016). Orthodontic treatment of malocclusion and its impact on oral health-related quality of life. Open Dent J.

[6] Bondemark L, Thilander B, Bondemark L, Bjerklin K (2018). Classification of malocclusions. Essential orthodontics.

[7] Caprioglio A, Fastuca R (2016). Étiologie et traitements des béances antérieures chez les patients en croissance: une étude narrative. Orthod Fr Décembre.

[8] Castilho RL, Matsumoto LH, Castilho GL, Weber SAT (2020). The interface between dentistry and respiratory sleep disorders in children. Sleep Sci.

[9] Graber T (1974). Orthodontics: Theory and Practice.

[10] Cozza P, Baccetti T, Franchi L, Mucedero M, Polimeni A (2007). Transverse features of subjects with sucking habits and facial hyperdivergency in the mixed dentition. Am J Orthod Dentofac Orthop.

[11] Deffrennes G, Deffrennes D (2017). Management of Brodie bite: note on surgical treatment. Int Orthod.

[12] Canut BJ (1992). Clinical orthodontics.

[13] Angle EH (1899). Classification of malocclusion. Dent Cosmos.

[14] Garde JB, Suryavanshi RK, Jawale BA, Deshmukh V, Dadhe DP, Suryavanshi MK (2014). An epidemiological study to know the prevalence of deleterious oral habits among 6 to 12 year old children. J Int Oral Health.

[15] Boj JR, Catalá M, García-Ballesta C, Mendoza A (2004). Early treatment of malocclusion. Pediatric dentistry.

[16] Kolawole KA, Folayan MO, Agbaje HO, Oyedele TA, Onyejaka NK, Oziegbe EO (2019). Oral habits and malocclusion in children resident in Ile-Ife Nigeria. Eur Arch Paediatr Dent.

[17] Arocha -rzuaga A, Aranda Godínez MS, Pérez Pérez Y, Granados Hormigó AE (2016). Malocclusions and deforming oral habits in school children with early mixed dentition. Medisan..

[18] Jiménez JJ (2016). Importance of atypical swallowing in malocclusions. Sanmarquina Dent.

[19] Giugliano D, Apuzzo F, Jamilian A, Perillo L (2015). Relationship between malocclusion and oral habits. Curr Res Dent.

[20] Lorente AA, Cortes O, Guzmán S, Vicente A, Garrido N (2019). Oral malocclusion and its relation to nutritive and non-nutritive habits in school children. Open J Dent Oral Med.

[21] Grippaudo C, Paolantonio EG, Antonini G, Saulle R, La Torre G, Deli R (2016). Association between oral habits, mouth breathing and malocclusion. Acta Otorhinolaryngol Ital.

[22] Paolantonio EG, Ludovici N, Saccomanno S, La Torre G, Grippaudo C (2019). Association between oral habits, mouth breathing and malocclusion in Italian preschoolers. Eur J Paediatr Dent.

[23] Fialho MPN, Pinzan-Vercelino CRM, Nogueira RP, Gurgel J de A. Relationship between facial morphology, anterior open bite and non-nutritive sucking habits during the primary dentition stage. Dent Press J Orthod. 2014;19:108–13. 10.1590/2176-9451.19.3.108-113.oar PMC4296632 25162574

[24] Pipa Vallejo A, Cuerpo García de los Reyes P, López-Arranz Monje E, González García M, Pipa Muñiz I, Acevedo Prado A. Prevalence of malocclusion in relation to non-nutritive sucking habits in children aged 3 to 9 years in Ferrol. Adv Dent. 2011;27(3):137–45.

[25] Omer MI, Abuaffan AH (2015). Prevalence of oral habits and its effect in primary dentition among Sudanese preschool children in Khartoum city. Indian J Dent Educ.

[26] Moimaz SAS, Garbin AJÍ, Lima AMC, Lolli LF, Saliba O, Garbin CAS (2014). Longitudinal study of habits leading to malocclusion development in childhood. BMC Oral Health..

[27] Condori LA, Mamani SLM (2016). Dentomaxillofacial alterations presented in patients with Hypertrophyroid Syndrome. Revista Científica Investigación Andina..

[28] Thomaz EBAF, Cangussu MCT, Assis AMO (2013). Malocclusion and deleterious oral habits among adolescents in a developing area in northeastern Brazil. Braz Oral Res.

[29] Aloufi SA, Jan HE, Abuhamda IS, Assiri AT, Samanodi HS, Alsulami AA (2017). Meta-analysis of prevalence of bad oral habits and relationship with prevalence of malocclusion. EC Dent Sci.

[30] Rossi RC, Rossi NJ, Rossi NJ, Yamashita HK, Pignatari SS (2015). Dentofacial characteristics of oral breathers in different ages: a retrospective case–control study. Prog Orthod.

[31] Farronato M, Lanteri V, Fama A, Maspero C (2020). Correlation between malocclusion and allergic rhinitis in pediatric patients: a systematic review. Children (Basel)..

[32] Katz CRT, Rosenblatt A, Gondim PPC (2004). Nonnutritive sucking habits in Brazilian children: effects on deciduous dentition and relationship with facial morphology. Am J Orthod Dentofac Orthop.

[33] D’Attilio M, Caputi S, Epifania E, Festa F, Tecco S (2005). Evaluation of cervical posture of children in skeletal class I, II, and III. Cranio.

